# Non-expanded adipose stromal vascular fraction cell therapy for multiple sclerosis

**DOI:** 10.1186/1479-5876-7-29

**Published:** 2009-04-24

**Authors:** Neil H Riordan, Thomas E Ichim, Wei-Ping Min, Hao Wang, Fabio Solano, Fabian Lara, Miguel Alfaro, Jorge Paz Rodriguez, Robert J Harman, Amit N Patel, Michael P Murphy, Roland R Lee, Boris Minev

**Affiliations:** 1 Medistem Inc, San Diego, CA, USA; 2 Department of Surgery, University of Western Ontario, London, Ontario, Canada; 3 Cell Medicine Institutes, San Jose, Costa Rica; 4 Hospital CIMA, San Jose, Costa Rica; 5 Cell Medicine Institutes, Panama City, Panama; 6 Vet-Stem, Inc. Poway, CA, USA; 7 Dept of Cardiothoracic Surgery, University of Utah, Salt Lake City, Utah, USA; 8 Division of Medicine, Indiana University School of Medicine, Indiana, USA; 9 Department of Radiology, University of Canlfornia San Diego, San Diego, CA, USA; 10 Veterans Administration, San Diego, CA, USA; 11 Moores Cancer Center, University of California, San Diego, CA, USA; 12 Department of Medicine, Division of Neurosurgery, University of California San Diego, San Diego, CA, USA

## Abstract

The stromal vascular fraction (SVF) of adipose tissue is known to contain mesenchymal stem cells (MSC), T regulatory cells, endothelial precursor cells, preadipocytes, as well as anti-inflammatory M2 macrophages. Safety of autologous adipose tissue implantation is supported by extensive use of this procedure in cosmetic surgery, as well as by ongoing studies using in vitro expanded adipose derived MSC. Equine and canine studies demonstrating anti-inflammatory and regenerative effects of non-expanded SVF cells have yielded promising results. Although non-expanded SVF cells have been used successfully in accelerating healing of Crohn's fistulas, to our knowledge clinical use of these cells for systemic immune modulation has not been reported. In this communication we discuss the rationale for use of autologous SVF in treatment of multiple sclerosis and describe our experiences with three patients. Based on this rationale and initial experiences, we propose controlled trials of autologous SVF in various inflammatory conditions.

## 1. Introduction

Adipose tissue has attracted interest as a possible alternative stem cell source to bone marrow. Enticing characteristics of adipose derived cells include: a) ease of extraction, b) higher content of mesenchymal stem cells (MSC) as compared to bone marrow, and c) ex vivo expandability of MSC is approximately equivalent, if not superior to bone marrow [1]. With one exception [2], clinical trials on adipose derived cells, to date, have been limited to ex vivo expanded cells, which share properties with bone marrow derived MSC [3-8]. MSC expanded from adipose tissue are equivalent, if not superior to bone marrow in terms of differentiation ability [9,10], angiogenesis stimulating potential [11], and immune modulatory effects [12]. Given the requirements and potential contaminations associated with ex vivo cellular expansion, a simpler procedure would be the use of primary adipose tissue derived cells for therapy. Indeed it is reported that over 3000 horses with various cartilage and bone injuries have been treated with autologous lipoaspirate fractions without cellular expansion [13]. In double blind studies of canine osteoarthritis statistically significant improvements in lameness, range of motion, and overall quality of life have been described [14,15].

If such approaches could be translated clinically, an easy-to-use autologous stem cell therapy could be implemented that is applicable to a multitude of indications. Indeed, this is the desire of commercial entities that are developing bench top closed systems for autologous adipose cell therapy, such as Cytori's Celution™ system [16] and Tissue Genesis' TGI 1000™ platform [17], which are presently entering clinical trials. Unfortunately, since the majority of scientific studies have focused on in vitro expanded adipose derived cells, relatively little is known about the potential clinical effects of the whole lipoaspirate that contains numerous cell populations besides MSC. From a safety perspective the process of autologous fat grafting has been commonly used in cosmetic surgery [18,19], so at least theoretically, autologous cell therapy, with the numerous cellular populations besides MSC that are found in adipose tissue, should be relatively innocuous. However, from an efficacy or disease-impact perspective, it is important to consider the various cellular components of adipose tissue and to develop a theoretical framework for evaluating activities that these components may mediate when administered systemically. For example, while attention is focused on the MSC component of adipose tissue, the high concentrations of monocytes/macrophages, and potential impact these may have on a clinical indication is often ignored.

In this paper we will discuss the potential use of the adipose derived cells for the treatment of inflammatory conditions in general, with specific emphasis on multiple sclerosis. Due to the chronic nature of the disease, the fact that in some situations remission naturally occurs, as well as lack of therapeutic impact on long term progression of current treatments, we examine the possibility of using autologous adipose derived cells in this condition. We will discuss the cellular components of adipose tissue, the biology of these components, how they may be involved in suppression of inflammatory/immunological aspects of MS, and conclude by providing case reports of three patients treatment with autologous adipose derived cells.

## 2. Components of Adipose Tissue

### Mesenchymal Stem Cells

The mononuclear fraction of adipose tissue, referred to as the stromal vascular fraction (SVF) was originally described as a mitotically active source of adipocyte precursors by Hollenberg et al. in 1968 [20]. These cells morphologically resembled fibroblasts and were demonstrated to differentiate into pre-adipocytes and functional adipose tissue in vitro [21]. Although it was suggested that non-adipose differentiation of SVF may occur under specific conditions [22], the notion of "adipose-derived stem cells" was not widely recognized until a seminal paper in 2001, where Zuk et al demonstrated the SVF contains large numbers of mesenchymal stem cells (MSC)-like cells that could be induced to differentiate into adipogenic, chondrogenic, myogenic, and osteogenic lineages [23]. Subsequent to the initial description, the same group reported after in vitro expansion the SVF derived cells had surface marker expression similar to bone marrow derived MSC, comprising of positive for CD29, CD44, CD71, CD90, CD105/SH2, and SH3 and lacking CD31, CD34, and CD45 expression [24]. Boquest et al characterized fresh CD45 negative, CD34 positive, CD105 positive SVF cells based on CD31 expression. They demonstrated that the CD31 negative cells exhibited mesenchymal properties and could be expanded in vitro, whereas the CD31 positive cells possessed endothelial-like properties with poor in vitro expansion capacity [25]. Mesenchymal cells with pluripotent potential have also been isolated from the liposuction aspirate fluid, which is the fluid portion of liposuction aspirates [26].

### Endothelial Progenitor Cells

In addition to MSC content, it was identified that SVF contains endothelial precursor cells (EPC). A common notion is that vasculature tissue continually replenishes damaged endothelial cells *de novo *from circulating bone marrow derived EPC [27], and that administration of exogenous EPC in animals having damaged vasculature can inhibit progression of atherosclerosis or restenosis [28,29]. Miranville et al demonstrated that human SVF cells isolated from subcutaneous or visceral adipose tissue contain a population of cells positive for CD34, CD133 and the drug efflux pump ABCG2 [30]. These cells had endothelial colony forming ability in vitro, and in vivo could induce angiogenesis in a hindlimb ischemia model. Interestingly, the concentrations of cells with the phenotype associated with in vivo angiogenic ability, CD31 negative and CD34 positive, was positively associated with body mass index. This suggests the possibility that endothelial precursor cell entrapment in adipose tissue of obese patients may be related to the reduced angiogenic function seen in obesity [31]. Several other groups have reported CD34 positive cells in the SVF capable of stimulating angiogenesis directly or through release of growth factors such as IGF-1, HGF-1 and VEGF [32-35]. The existence of a CD34 positive subset in the SVF may indicate possibility of cells with not only endothelial but also hematopoietic potential. Indeed at least one report exists of a bipotent hematopoietic and angiopoietic phenotype isolated from the SVF [36]. Thus from these data it appears that SVF contains at least 2 major populations of stem cells, an MSC compartment and an EPC compartment that may have some hematopoietic activity. When these cells are quantified, one author describes that from primary isolated SVF, approximately 2% of the cells have the hematopoietic-associated CD34+ CD45+ phenotype, and 6.7% having a mesenchymal CD105+ CD146+ phenotype [37]. Many studies using SVF perform in vitro expansion of the cells, this causes selection for certain cell populations such as MSC and decreases the number of CD34 cells [38]. Thus in vitro expanded SVF derived cells can not be compared with primary isolated SVF cells.

### Immune Regulatory Monocytes/Macrophages

In addition to its stem/progenitor cell content, the SVF is known to contain monocytes/macrophages. Although pluripotency of monocytic populations has previously been described [39,40], we will focus our discussion to immunological properties. Initial experiments suggested that macrophage content of adipose tissue was associated with the chronic low grade inflammation found in obese patients. This was suggested by co-culture experiments in which adipocytes were capable of inducing TNF-alpha secretion from macrophage cell lines in vitro [41]. Clinical studies demonstrated that adipocytes also directly release a constitutive amount of TNF-alpha and leptin, which are capable of inducing macrophage secretion of inflammatory mediators [42]. It appears from several studies in mice and humans that when monocytes/macrophages are isolated from adipose tissue, they in fact possess anti-inflammatory functions characterized by high expression of IL-10 and IL-1 receptor antagonist [43-45]. These adipose derived macrophages have an "M2" phenotype, which physiologically is seen in conditions of immune suppression such as in tumors [46], post-sepsis compensatory anti-inflammatory syndrome [47,48], or pregnancy associated decidual macrophages [49]. It is estimated that the monocytic/macrophage compartment of the SVF is approximately 10% based on CD14 expression [37]. Interestingly, administrations of ex vivo generated M2 macrophages have been demonstrated to inhibit kidney injury in an adriamycin-induced model [50]. In the context of MS, alternatively activated, M2-like microglial cells are believed to inhibit progression in the EAE model [51]. Thus the anti-inflammatory activities of M2 cells are a potential mechanism of therapeutic effect of SVF cells when isolated from primary sources and not expanded.

### T Regulatory Cells

It has been reported by us and others, that activation of T cells in the absence of costimulatory signals leads to generation of immune suppressive CD4+ CD25+ T regulatory (Treg) cells [52,53]. Thus local activation of immunity in adipose tissue would theoretically be associated with reduced costimulatory molecule expression by the M2 macrophages, which theoretically may predispose to Treg generation. Conversely, it is known that Tregs are involved in maintaining macrophages in the M2 phenotype [54]. Supporting the possibility of Treg in adipose tissue also comes from the high concentration of local MSC which are known to secrete TGF-beta [55] and IL-10 [56], both involved in Treg generation [57]. Indeed numerous studies have demonstrated the ability of MSC to induce Treg cells [56,58-60]. To test the possibility that Treg exist in the SVF, we performed a series of experiments isolating CD4, CD25 positive cells from the SVF of BALB/c mice and compared frequency between other tissues, (lymph node and spleen). We observed a 3 fold increase in the CD4+, CD25+ compartment as compared to control tissues. Functionally, these cells were capable of suppressing ConA stimulated syngeneic CD4+ CD25+ negative cells (*manuscript in preparation*).

## 3. Treatment of Autoimmunity with Adipose Cells

In general, MSC, whether derived from the bone marrow, adipose, or other sources, have been demonstrated to exert dual functions that are relevant to autoimmunity [61-65]. These conditions are usually exemplified by activation of innate immune components, breakdown of self tolerance of the adaptive immune response, and subsequent destruction of tissues. Although these are generalizations, an initial insult either by foreign microorganisms, or other means, causes tissue damage and activation of innate immunity, which under proper genetic background leads to re-activation/escape from anergy of "self"-recognizing T cell clones, thus causing more tissue damage, activation of immunity, and lose of function. MSC inhibit innate immune activation by blocking dendritic cell maturation [66,67], by suppressing macrophage activation [68], and by producing agents such as IL-1 receptor antagonist [69] and IL-10 [70] that directly block inflammatory signaling. Perhaps the strongest example of MSC inhibiting the innate immune response is the recent publication of Nemeth et al, which demonstrated that administration of MSC can block onset of sepsis in the aggressive cecal ligation and puncture model [68]. Through inhibiting DC activation, MSC suppress subsequent adaptive immunity by generating T regulatory (Treg) cells [59], as well as blocking cytotoxic activities of CD8 cells. In some situations, increased immunoregulatory activity is reported with expanded MSC compartment of SVF as reported by Mcintosh et al. [71].

In addition to inhibiting pathological innate and adaptive immunity, MSC have the ability to selectively home to areas of tissue damage, and mediate direct or indirect repair function. As an example, CXCR-4 expression of MSC allows homing toward injured/hypoxic tissue after intravenous administration. Indeed this has allowed for numerous studies demonstrating positive effects of intravenously administered MSC causing regeneration in many tissues such as CNS injury [72,73], transplant rejection [59], toxin-induced diabetes [74], nephropathy [75], and enteropathy [76]. The regenerative effects of MSC have been postulated to be mediated by differentiation into damaged tissue, although this is somewhat controversial, as well as through secretion of growth factors/antiapoptotic factors which induce tissue regeneration [77,78].

The ability of MSC to inhibit immune response, while offering the possibility of inducing/accelerating healing of tissue that has already been damaged, makes this population attractive for treatment of autoimmune disorders. While numerous studies clinical studies are using expanded MSC derived from the bone marrow [79-81], here we chose an indication of autologous adipose SVF based on the immunological profile, the length of disease progress allowing several interventions, and the fact that the disease naturally has periods of remission during which the rationale would be to amplify a process that already is underway.

## 4. Multiple Sclerosis

Multiple sclerosis (MS) is an autoimmune condition in which the immune system attacks the central nervous system (CNS), leading to demyelination. It may cause numerous physical and mental symptoms, and often progresses to physical and cognitive disability. Disease onset usually occurs in young adults, and is more common in women [82]. MS affects the areas of the brain and spinal cord known as the white matter. Specifically, MS destroys oligodendrocytes, which are the cells responsible for creating and maintaining the myelin sheath, which helps the neurons carry electrical signals. MS results in a thinning or complete loss of myelin and, less frequently, transection of axons [83].

Current therapies for MS include steroids, immune suppressants (cyclosporine, azathioprine, methotrexate), immune modulators (interferons, glatiramer acetate), and immune modulating antibodies (natalizumab). At present none of the MS treatment available on the market selectively inhibit the immune attack against the nervous system, nor do they stimulate regeneration of previously damaged tissue.

### Treg cells modulate MS

Induction of remission in MS has been associated with stimulation of T regulatory cells. For example, patients responding to the clinically used immune modulatory drug glatiramer acetate have been reported to have increased levels of CD4+, CD25+, FoxP3+ Treg cells in peripheral blood and cerebral spinal fluid [84]. Interferon beta, another clinically used drug for MS induces a renormalization of Treg activity after initiation of therapy through stimulation of de novo regulatory cell generation [85]. In the animal model of MS, experimental allergic encephalomyelitis (EAE), disease progression is exacerbated by Treg depletion [86], and natural protection against disease in certain models of EAE is associated with antigen-specific Treg [87]. Thus there is some reason to believe that stimulation of the Treg compartment may be therapeutically beneficial in MS.

### Endogenous neural stem cells affect MS recovery

In addition to immune damage, MS patients are known to have a certain degree of recovery based on endogenous repair processes. Pregnancy associated MS remission has been demonstrated to be associated with increased white matter plasticity and oligodendrocyte repair activity [88]. Functional MRI (fMRI) studies have suggested that various behavioral modifications may augment repair processes at least in a subset of MS patients [89]. Endogenous stem cells in the sub-ventricular zone of brains of mice and humans with MS have been demonstrated to possess ability to differentiate into oligodendrocytes and to some extent assist in remyelination [89]. For example, an 8-fold increase in de novo differentiating sub-ventricular zone derived cells was observed in autopsy samples of MS patients in active as compared to non-active lesions [90].

### Stem Cell Therapy for MS

The therapeutic effects of MSC in MS have been demonstrated in several animal studies. In one of the first studies of immune modulation, Zappia et al. demonstrated administration of MSC subsequent to immunization with encephalomyelitis-inducing bovine myelin prevented onset of the mouse MS-like disease EAE. The investigators attributed the therapeutic effects to stimulation of Treg cells, deviation of cytokine profile, and apoptosis of activated T cells [73]. It is interesting to note that the MSC were injected intravenously. Several other studies have shown inhibition of EAE using various MSC injection protocols [91,92].

To our knowledge there is only one publication describing clinical exploration of MSC in MS. An Iranian group reported using intrathecal injections of autologous culture expanded MSC in treatment unresponsive MS patients demonstrated improvement in one patient (EDSS score from 5 to 2.5), no change in 4 patients, and progressive disease in 5 patients based on EDSS score. Functional system assessment revealed six patients had improvement in their sensory, pyramidal, and cerebellar functions. One showed no difference in clinical assessment and three deteriorated [93].

## 5. Case Reports

Given the rationale that autologous SVF cells have a reasonable safety profile, and contain both immune modulatory and regenerative cell populations, a physician-initiated compassionate-use treatment was explored in 3 patients. Here we describe their treatments and histories.

### #CR-231

In 2005, a 50-year-old man was diagnosed with Relapsing-remitting MS, presenting with tonic spasms, stiffness, gait imbalance, excessive hearing loss, loss of coordination, numbness in both feet, sexual dysfunction, severe pain all over his body, fatigue and depression. In 2005, the patient experienced refractory spells of tonic flexion spasms, occurring for several minutes at a time and multiple times throughout the day. He was treated with muscle relaxants, I.V. steroids and Tegretol, and his condition had improved. However, in 2006 he experienced severe uncontrollable tonic extensions of all four extremities lasting about two minutes and associated with significant pain. Cranial MRI done at that time revealed at least 30 periventricular white matter lesions. Patient also reported excellent response to Solu-Medrol infusions. Therefore, the combination of response to steroids, characteristic MRI abnormalities and positive oligoclonal banding strongly suggested a diagnosis of Relapsing Remitting MS. Infusions of Tysabri (Natalizumab, Biogen Idec) every four weeks were prescribed in November 2006, with excellent results and no significant side effects. However, in March 2007 patient reported spasticity approximately three weeks after the infusions, leading to alteration of his Tysabri infusion regimen to Q3 weeks. By June 2007 the patient had began complaining of significant memory loss and by September 2007 he has had recurrence of his tonic spasms with multiple attacks daily. He was treated with Solu-Medrol, Baclofen, Provigil, Tegretol, Trileptal, Tysabri, Vitamins, Omega-3 and Zanaflex with some improvement of his neurologic symptoms. However, he complained of severe abdominal pain, decreased appetite and melanotic stools, consistent with stress ulcer secondary to steroid treatment. By November 2007 the patient was still somewhat responsive to Tysabri and I.V. Solu-Medrol, but continued to experience multiple severe tonic spasms at a rate of 30 – 40 spasms per month.

**Figure 1:**
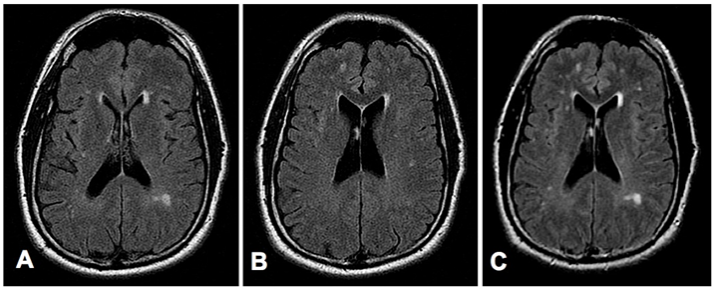
**MRI Images obtained before (Panels A and B), and six months after (Panel C) the stem cell treatment of patient 1**. **Panels A and B**: Consecutive axial FLuid-Attenuated Inversion Recovery (FLAIR) images through the lateral ventricles show multiple small foci of bright signal in the periventricular and subcortical white matter, consistent with plaques of multiple sclerosis. **Panel C**: Axial FLAIR image shows no significant change in the multiple periventricular and subcortical white-matter plaques. (For the comparison, note that this slice is positioned between those in A and B, and at slightly different scanning-angle, so it includes lesions of both those slices, as well as others slightly out-of their plane.).

In May 2008, the patient was treated with two I.V. infusions of 28 million SVF cells and multiple intrathecal and intravenous infusions of allogeneic CD34+ and MSC cells. MSC were third party unmatched and CD34 were matched by mixed lymphocyte reaction. Infusions were performed within a 9-day period and were very well tolerated without any adverse or side effects. No other treatments were necessary during the patient's stay. After the second stem cell infusion the patient reported a significant decrease of his generalized pain. However, he continued to experience severe neck and shoulder pain and was re-evaluated by his neurologist. Two months after the stem cell therapy, the volume of his hearing aids had to be lowered once per week over 4 weeks. Three months after the stem cell infusions the patient reported a significant improvement of his cognition and almost complete reduction of the spasticity in his extremities. He mentioned that he has had 623 tonic seizures in the past and confirmed that he has not experienced any more seizures since the completion of the stem cell therapy. A neurological evaluation performed three months after the stem cell infusions revealed an intact cranial nerve (II-XII) function and no nystagmus, normal motor function without any atrophy or fasciculations, and intact sensory and cerebellar functions and mental status. New MRI images, obtained 6 months after the stem cell treatment showed lesions, very similar to the lesions observed before the stem cell treatment (Figure 1). The patient also reported significantly improved memory, sexual function, and energy level. Currently, the patient is taking only multivitamin, minerals and Omega 3.

### #233

Second patient: A 32-year-old man was diagnosed in 2001 with relapsing-remitting MS, presenting with fatigue and depression, uneven walk pattern, cognitive dysfunction, and a progressive decline in his memory without any specific neurological symptoms. In 2002 he was started on weekly intramuscular Avonex (IFN-b1a, Biogen Idec) and has had no further exacerbations and no evidence of progressive deterioration. Patient's fatigue was treated well with Provigil, and his mood improved significantly due to treatment with Wellbutrin SR. In 2007, the patient complained of some mood changes, with more agitation, irritability, mood destabilization, and cognitive slowing. As depression was suspected in playing a central role in patient's condition, Razadyne was added to the antidepressant regimen.

In 2008, the patient was treated with two I.V. infusions of 25 million autologous adipose-derived SVF cells and multiple intrathecal and intravenous infusions of allogeneic CD34+ and MSC cells. MSC were third party unmatched and CD34 were matched by mixed lymphocyte reaction. All infusions were performed within a 10-day period and were very well tolerated without any significant side effects. The treatment plan also included physical therapy sessions.

**Figure 2:**
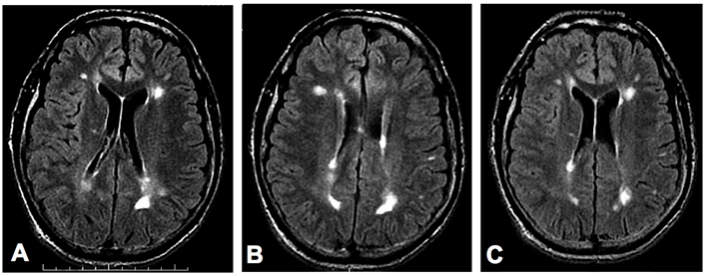
**MRI Images obtained before (Panels A and B), and seven months after (Panel C) the stem cell treatment of patient 2**. **Panels A and B**: Consecutive axial FLuid-Attenuated Inversion Recovery (FLAIR) images through the lateral ventricles show multiple small patches of bright signal in the periventricular and subcortical white matter, consistent with plaques of multiple sclerosis. **Panel C**: Axial FLAIR image shows no significant change in the multiple periventricular and subcortical white-matter plaques. (For the comparison, note that this slice is positioned similar to slice A but at slightly different scanning-angle, so it includes lesions of both slices A and B.).

Three months after the stem cell infusions the patient reported a significant improvement of his balance and coordination as well as an improved energy level and mood. New MRI images, obtained 7 months after the stem cell treatment showed lesions, very similar to the lesions observed before the stem cell treatment (Figure 2). Currently, he is not taking any antidepressants and is reporting a significantly improved overall condition. His current treatment regiment includes a weekly injection of Avonex, vitamins, minerals and Omega 3.

### #255

The patient was diagnosed with relapsing-remitting MS in 1993, presenting symptoms were noticeable tingling and burning sensation in the right leg, followed by paraplegia lasting almost three weeks. Neurological investigations at the time uncovered MRI findings suggestive for a demyelinating syndrome. In June of 2008, the patient was treated with two I.V. infusions of 75 million autologous adipose-derived SVF cells and multiple intrathecal and intravenous infusions of allogeneic CD34+ and MSC cells. MSC were third party unmatched and CD34 were matched by mixed lymphocyte reaction. All infusions were performed within a 10-day period and were very well tolerated without any significant side effects. His gait, balance and coordination improved dramatically oven a period of several weeks. His condition continued to improve over the next few months and he is currently reporting a still continuing improvement and ability to jog, run and bike for extended periods of time daily.

## Conclusion

The patients treated were part of a compassionate-use evaluation of stem cell therapeutic protocols in a physician-initiated manner. Previous experiences in MS patients using allogeneic CD34+ cord blood cells together with MSC did not routinely result in substantial improvements observed in the three cases described above. While obviously no conclusions in terms of therapeutic efficacy can be drawn from the above reports, we believe that further clinical evaluation of autologous SVF cells is warranted in autoimmune conditions.

## Competing interests

Thomas E Ichim and Neil H Riordan are management and shareholders of Medistem Inc, a company that has filed intellectual property on the use of adipose stromal vascular fraction cells for immune modulation.

## Authors' contributions

All authors read and approved the final manuscript. NHR, TEI, WPM, HW, FS, FL, MA, JPR, RJH, ANP, MPM, RRL and BM conceived experiments, interpreted data, and wrote the manuscript.
